# Bioinformatics analysis to identify potential biomarkers and therapeutic targets for ST-segment–elevation myocardial infarction-related ischemic stroke

**DOI:** 10.3389/fneur.2022.894289

**Published:** 2022-08-11

**Authors:** Shuo Feng, Rui Li, Qingqing Zhou, Fengling Qu, Wei Hu, Xinfeng Liu

**Affiliations:** Stroke Center and Department of Neurology, The First Affiliated Hospital of USTC, Division of Life Sciences and Medicine, University of Science and Technology of China, Hefei, China

**Keywords:** gene analysis, biomarker, ST-segment-elevation myocardial infarction, ischemic stroke, therapeutic targets

## Abstract

**Background:**

Acute myocardial infarction (AMI) is one of the major causes of mortality and disability worldwide, and ischemic stroke (IS) is a serious complication after AMI. In particular, patients with ST-segment–elevation myocardial infarction (STEMI) are more susceptible to IS. However, the interrelationship between the two disease mechanisms is not clear. Using bioinformatics tools, we investigated genes commonly expressed in patients with STEMI and IS to explore the relationship between these diseases, with the aim of uncovering the underlying biomarkers and therapeutic targets for STEMI-associated IS.

**Methods:**

Differentially expressed genes (DEGs) related to STEMI and IS were identified through bioinformatics analysis of the Gene Expression Omnibus (GEO) datasets GSE60993 and GSE16561, respectively. Thereafter, we assessed protein-protein interaction networks, gene ontology term annotations, and pathway enrichment for DEGs using various prediction and network analysis methods. The predicted miRNAs targeting the co-expressed STEMI- and IS-related DEGs were also evaluated.

**Results:**

We identified 210 and 29 DEGs in GSE60993 and GSE16561, respectively. CD8A, TLR2, TLR4, S100A12, and TREM1 were associated with STEMI, while the hubgenes, IL7R, CCR7, FCGR3B, CD79A, and ITK were implicated in IS. In addition, binding of the transcripts of the co-expressed DEGs MMP9, ARG1, CA4, CRISPLD2, S100A12, and GZMK to their corresponding predicted miRNAs, especially miR-654-5p, may be associated with STEMI-related IS.

**Conclusions:**

STEMI and IS are related and MMP9, ARG1, CA4, CRISPLD2, S100A12, and GZMK genes may be underlying biomarkers involved in STEMI-related IS.

## Introduction

Acute myocardial infarction (AMI) is a leading cause of disability and mortality worldwide, and ischemic stroke (IS) is a serious complication after AMI (1). Complex IS can cause significant pain and financial burden to patients, and the rate of mortality is two times higher in comparison with patients only experiencing AMI (1, 2). Pathophysiological mechanisms and common risk factors, including age, hypertension, and diabetes mellitus, are similar in cardiovascular and cerebrovascular diseases (3). The incidence of post-AMI strokes can be improved by providing more therapies for vascular risk factors, including treatments for diabetes mellitus and hypertension, lipid-lowering treatments, and reperfusion with PCI (4). Compared with other types of AMI, patients with ST-segment–elevation myocardial infarction (STEMI) have a more increased risk of IS (5–7). Guptill's group also showed that there was a relative long-term risk of IS in patients with STEMI treated with percutaneous coronary intervention (PCI) (8). Whereas there have been few studies of the prevalence and clinical outcomes associated with acute IS in patients with AMI, and existing studies have had small sample sizes and reported contrasting results (9–12). To better diagnose and treat IS after AMI, new biomarkers and therapeutic targets need to be identified. Bioinformatics analysis has been widely employed in exploring novel biomarkers for neurological disease (13) and cardiovascular disease (14). In this study, we identified co-expressed differentially expressed genes (co-DEGs) in STEMI and IS transcription data from GEO to clarify the molecular mechanisms and pathophysiology of STEMI-related DEGs (STEMI-DEGs) and IS-related DEGs (IS-DEGs). Moreover, we predicted microRNAs (miRNAs) specific for patients with STEMI prone to IS, which may serve as underlying biomarkers or therapeutic targets for STEMI-IS.

## Methods

### Materials and methods

Microarray data “Series Matrix File(s)” for GSE60993, GSE16561, and GSE60319 were downloaded from GEO (https://www.ncbi.nlm.nih.gov/geo/) and were generated using GPL6884, GPL6883, and GPL19071 (15). GSE60993 contains data from blood samples from 26 patients with acute coronary syndrome (7 patients with STEMI, 10 patients with non-STEMI, and 9 patients with unstable angina) and 7 normal controls. GSE16561 includes blood samples from 39 patients with IS and 24 healthy controls. The STEMI group and normal controls in GSE60993 and the IS group and healthy controls in GSE16561 were selected to explore potential biomarkers. A miRNA expression profile, GSE60319 (40 patients with IS and 10 controls), was then used for subsequent miRNA-mRNA network analysis.

### DEG analysis

Before identifying DEGs, we performed boxplot analysis to evaluate the expression level of samples in each dataset and then used the normalize BetweenArrays function in the “limma” package of R to exclude batch effect. The criteria for selecting DEGs were |log2FC|>1.0 and false discovery rate (FDR) <0.05; the criteria for differentially expressed miRNAs (DE-miRNAs) were |log2FC| > 2.5 and *P*-value <0.05 to identify more important DE-miRNAs. The inverse of the total gene number (0.0006035) was less than the lowest *P*-value (0.0009911013) in GSE60319; hence, the adjusted *P*-values were unreliable. Probes matching multiple genes were removed. Volcanoplots and heatmaps were applied to visualize the DEGs in the downloaded datasets. A Venn diagram was constructed to show co-DEGs for STEMI and IS using Funrich (http://funrich.org/).

### Interaction networks and functional analysis

DAVID (https://david.ncifcrf.gov/) was applied to perform Gene Ontology (GO) and Kyoto Encyclopedia of Genes and Genomes (KEGG) pathway enrichment analyses of STEMI- and IS-DEGs (16). KEGG pathways and GO biological function terms with a *P*-value < 0.05 were considered to be significantly enriched, and annotation visualization, as well as integrated discovery, was supplemented using REACTOME with the following criteria: *P*-value < 0.05 and count ≥ 5 (v77; http://www.reactome.org) (17). We used Cytoscape (v3.8.2; http://cytoscape.org/) to visualize the protein-protein interaction (PPI) networks and node degrees constructed by STRING (v11.5; http://string-db.org) (18), with the criterion confidence score >0.4.

In addition, AmiGO (v2.0; http://amigo.geneontology.org/amigo/) was employed to further verify the accuracy of the identified co-DEGs and annotate biological functions (19). TargetScan (v7.2; http://www.targetscan.org/vert_72/) (20), mirWalk (http://mirwalk.umm.uni-heidelberg.de/) (21), and mirDIP (http://ophid.utoronto.ca/mirDIP/) (22) were applied to predict miRNAs targeting co-DEGs. GO and KEGG enrichment analyses based on the selected miRNAs were conducted using Diana-miRPath (v3.0; http://www.microrna.gr/miRPa) (23).

### Identification of co-DEGs related to nervous or cardiovascular diseases

The Comparative Toxicogenomics Database (http://ctdbase.org/) was employed to identify novel relationships between co-DEGs and cardiovascular diseases or nervous system diseases by calculating prediction scores (24).

## Results

### DEGs in STEMI and IS

**Figure 1:**
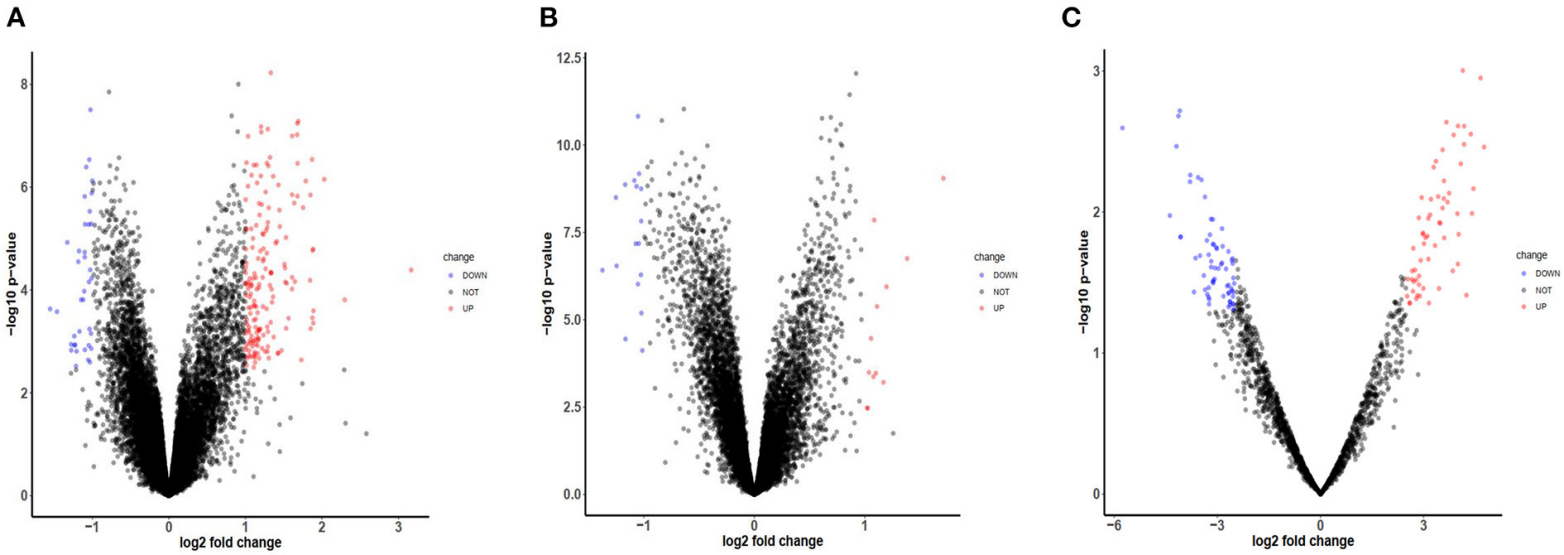
Volcano plots of mRNA and miRNA expression in GEO datasets. **(A)** The volcano plot of GSE-STEMI (GSE60993). **(B)** The volcano plot of GSE-IS (GSE16561). **(C)** The volcano plot of mi-GSE-IS (GSE60319).

**Figure 2:**
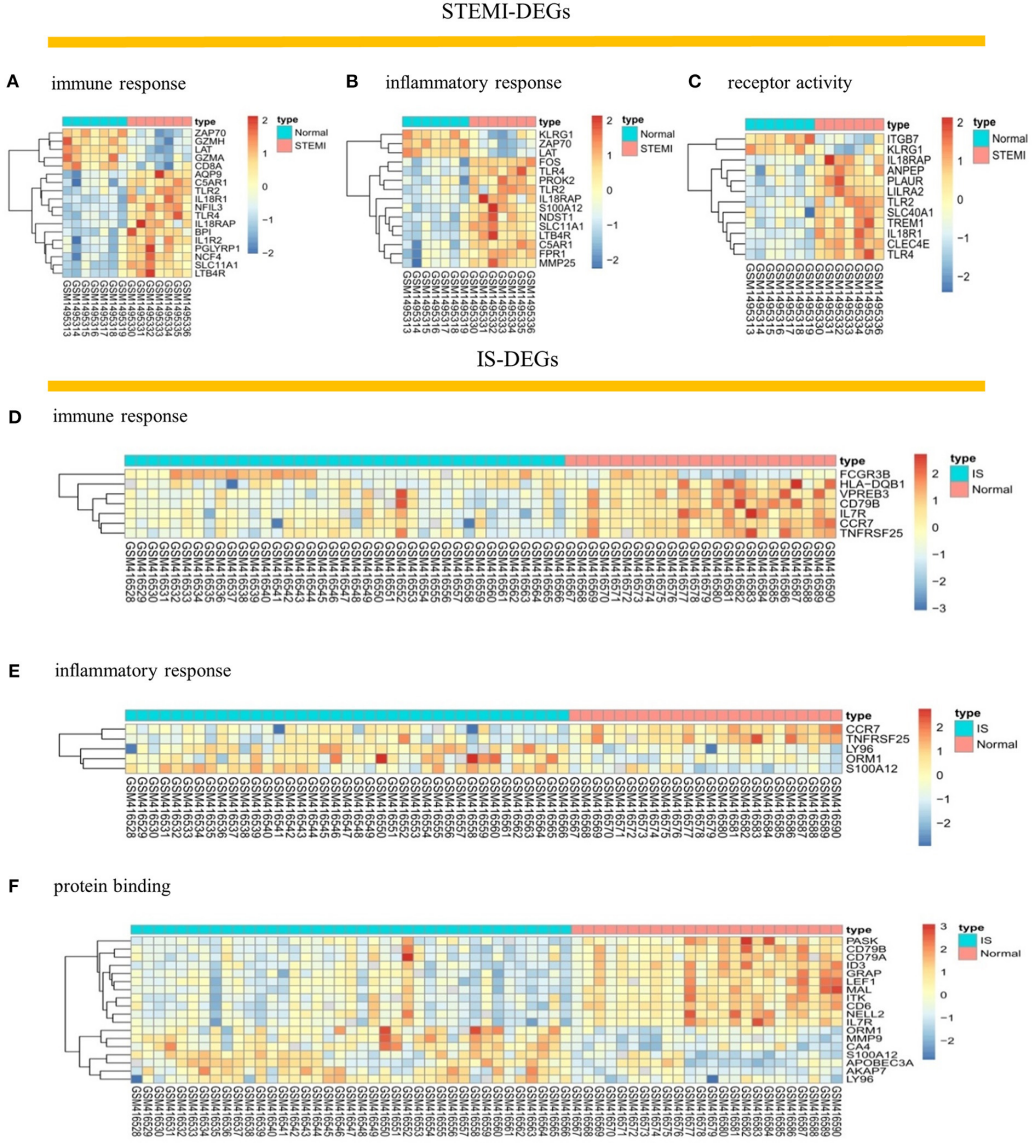
Visualization of STEMI- and IS-DEGs expression with heatmaps. **(A–C)** STEMI-DEGs related to immune response, inflammatory response, and receptor activity. **(D–F)** IS-DEGs related to immune response, inflammatory response, and protein binding. Red: high expression, blue: low expression.

After checking the quality of the data (Supplementary Figure 1), we identified 210 DEGs (172 upregulated and 38 downregulated) in GSE60993 and 29 DEGs (12 upregulated and 17 downregulated) in GSE16561 (Figures 1A,B, Supplementary Table 1). Expression heatmaps of STEMI-DEGs associated with immune and inflammatory responses and receptor activity are shown in Figures 2A–C. Figures 2D–F show the gene expression values of IS-DEGs related to immune response, inflammatory response, and protein binding.

### Analysis of PPI network, functional GO terms and pathway enrichment analyses

**Figure 3:**
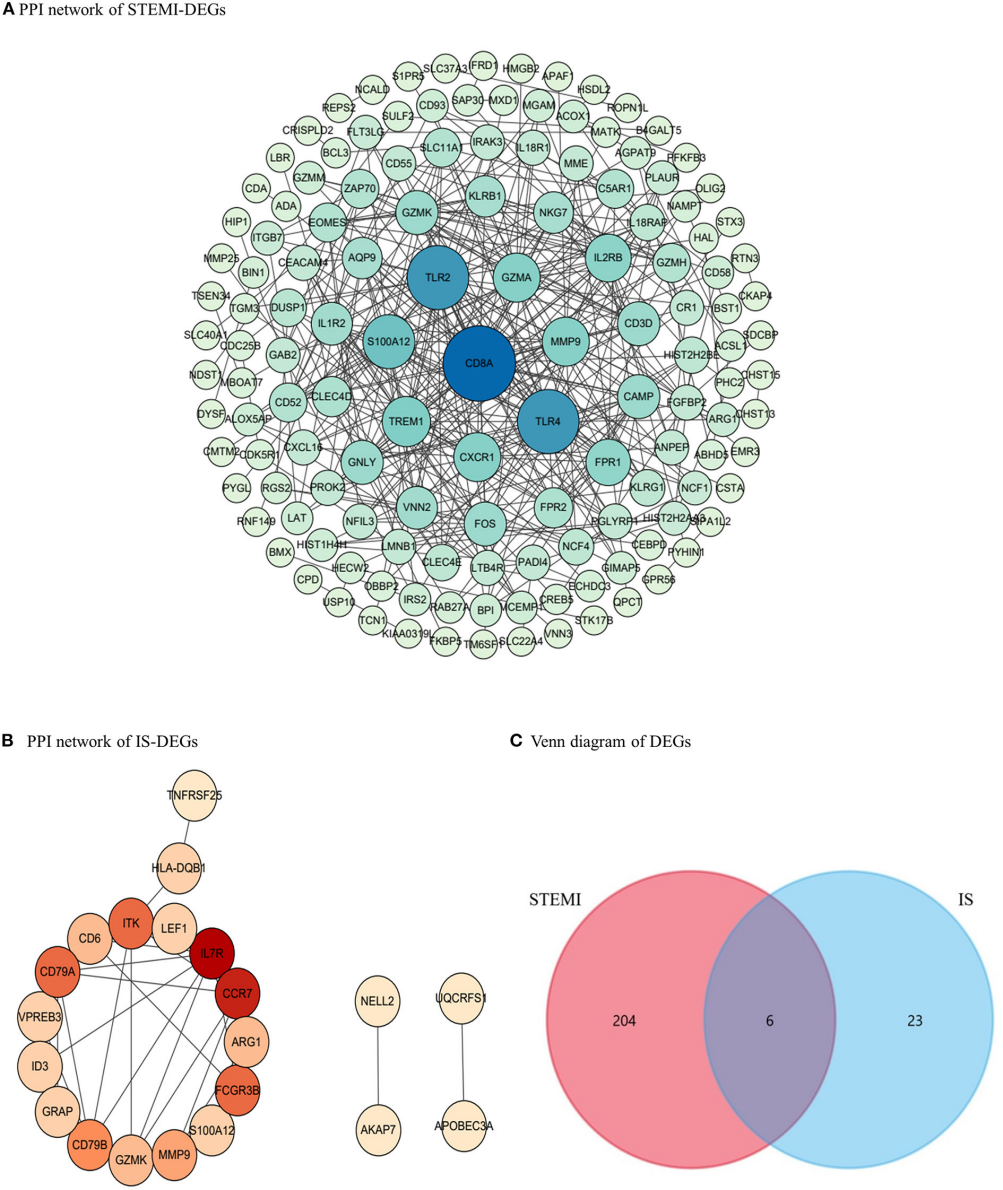
PPI networks and the Venn diagram. **(A)** PPI network for STEMI-DEGs. Blue, the greater degree; green, the lower degree **(B)** PPI network for IS-DEGs. Red, the greater degree; yellow, the lower degree. **(C)** The Venn diagram showing co-DEGs specific to STEMI-related IS.

We identified 139 and 21 nodes from the PPI networks for the STEMI- and IS-DEGs, respectively (Figures 3A,B). The hub nodes, including CD8a molecule (CD8A, degree = 38), toll-like receptor 2 (TLR2, degree = 29), toll-like receptor 4 (TLR4, degree = 29), S100 calcium-binding protein A12 (S100A12, degree = 21), and triggering receptor expressed on myeloid cells 1 (TREM1, degree = 18), were considered to be hubgenes in the STEMI network. However, in the IS network, the hubgenes, interleukin 7 receptor (IL7R, degree = 9), C-C motif chemokine receptor 7 (CCR7, degree = 8), Fc fragment of IgG receptor IIIb (FCGR3B, degree = 6), CD79a molecule (CD79A, degree = 6), and IL2 inducible T cell kinase (ITK, degree = 6) had relatively higher degrees.

**Figure 4:**
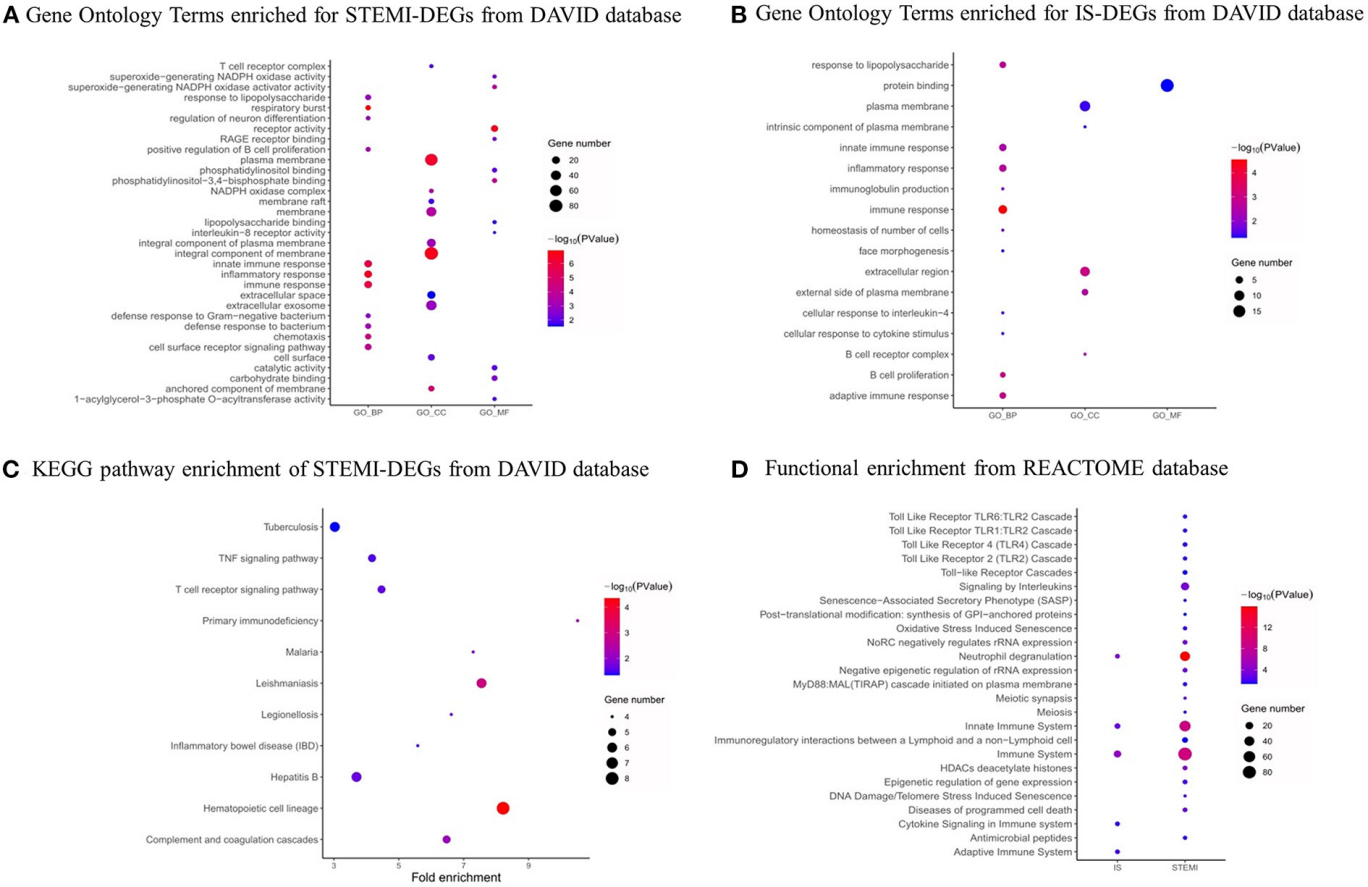
GO functional and pathway analysis. **(A)** GO functional analysis of STEMI-DEGs. **(B)** GO functional analysis of IS-DEGs. **(C)** KEGG pathway analysis of STEMI-related DEGs. **(D)** REACTOME pathway analysis of STEMI- and IS-related DEGs. Dot sizes represent counts of enriched DEGs, and dot colors represent negative log10 (P) values. Red: higher expression, blue: lower expression.

We used the DAVID database to conduct GO and KEGG analysis. As shown in Figures 4A,B, the top five GO biological process (BP) terms associated with STEMI-DEGs were respiratory burst (*p*-value: 7.14E-08), immune response (*p*-value: 3.01E-07), innate immune response (*p*-value: 4.05E-07), inflammatory response (*p*-value: 9.80E-06), and defense response to bacterium (*p*-value: 3.67E-04). The significantly enriched cellular component (CC) terms were an anchored component of membrane (*p*-value: 7.96E-06), plasma membrane (*p*-value: 1.09E-04), NADPH oxidase complex (*p*-value: 1.46E-04), an integral component of membrane (*p*-value: 1.60E-04), and membrane (*p*-value: 9.50E-04). The following terms were found to be enriched in molecular function (MF):receptor activity (*p*-value: 3.84E-06), phosphatidylinositol-3,4-bisphosphate binding (*p*-value: 6.55E-05), superoxide-generating NADPH oxidase activator activity (*p*-value: 7.88E-05), protein heterodimerization activity (*p*-value: 0.003), and RAGE receptor binding (*p*-value: 0.004). With respect to IS-DEGs, BP terms associated with immune response (*p*-value: 2.83E-05), B cell proliferation (*p*-value: 0.001), adaptive immune response (*p*-value: 0.001), response to lipopolysaccharide (*p*-value: 0.002), and inflammatory response (*p*-value:0.002) were significantly enriched. For CC, the significant enrichment was observed for the extracellular region (*p*-value: 0.001), the external side of plasma membrane (*p*-value: 0.003), B cell receptor complex (*p*-value: 0.004), the intrinsic component of the plasma membrane (*p*-value: 0.038), and the plasma membrane (*p*-value: 0.039). For MF, protein binding was enriched (*p*-value: 0.049). The results of KEGG pathway analysis are shown in Figure 4C. STEMI-DEGs were mainly enriched in pathways, including hematopoietic cell lineage (*p*-value: 4.38E-05), leishmaniasis (*p*-value: 0.001), primary immunodeficiency (*p*-value: 0.006), complement and coagulation cascades (*p*-value: 0.007), and malaria (*p*-value: 0.017). There were no significant KEGG pathways enriched for IS-DEGs. Some additional associations were detected when using the REACTOME database to conduct GO term enrichment analysis (Figure 4D).

**Figure 5:**
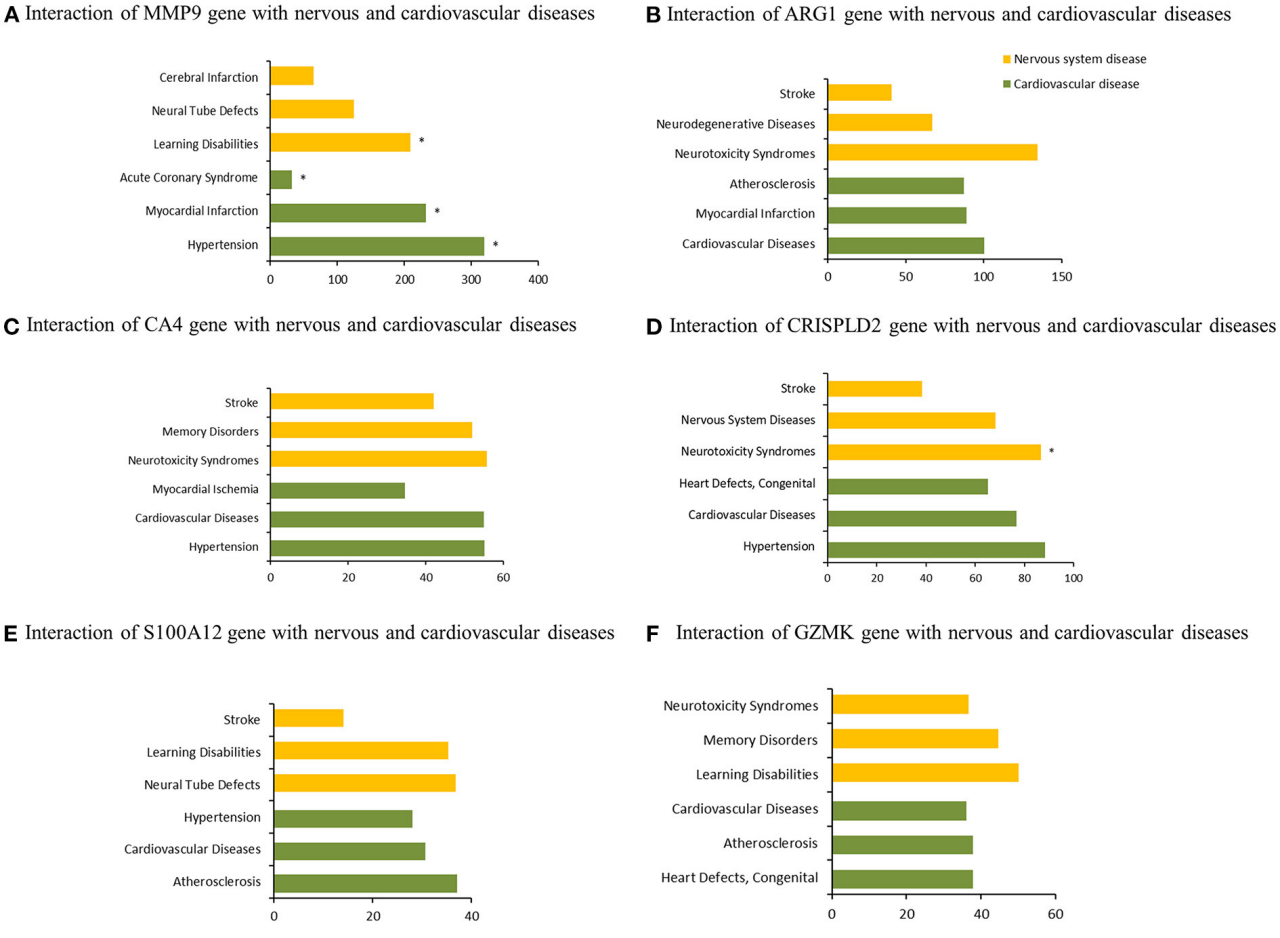
Association of co-DEGs with nervous system and cardiovascular diseases. *indicates direct evidence of involvement in this disease.

Six co-DEGs were observed, namely, matrix metallopeptidase 9 (MMP9), arginase 1 (ARG1), carbonic anhydrase 4 (CA4), the cysteine-rich secretory protein LCCL domain containing 2 (CRISPLD2), S100 calcium-binding protein A12 (S100A12), and granzyme K (GZMK) (Figure 3C, Supplementary Table 1). The AmiGO database was employed to further verify the accuracy of the identified co-DEGs and annotate their biological functions (Table 1). The analysis of the Comparative Toxicogenomics Database illustrated that co-DEGs were associated with several nervous system and cardiovascular diseases (Figure 5).

**Table 1:** GO terms of co-expressed genes specific for STEMI-related ischemic stroke.

**Gene/product**	**GO class (direct)**	**Evidence**	**Evidence with**	**Reference**
MMP9	Response to hypoxia	IEP		PMID:17289933
	Regulation of neuroinflammatory response	TAS		PMID:25049354
	Negative regulation of glial cell proliferation	IMP	ZFIN:ZDB-FISH-210714-2	PMID:32034934
	Positive regulation of angiogenesis	ISO	RGD:621320	MGI:MGI:4417868
	Heart development	ISO	RGD:621320	MGI:MGI:4417868
	Positive regulation of vascular associated smooth muscle cell proliferation	IMP		PMID:18667463
	Extracellular matrix organization	IBA	PANTHER:PTN001303987	PMID:21873635
	Positive regulation of apoptotic process	IEA	UniProtKB:P41245	GO_REF:0000107
ARG1	Immune system process	IEA	UniProtKB-KW:KW-0391	MGI:MGI:1354194
	Cellular response to transforming growth factor beta stimulus	IEA	UniProtKB:P07824	GO_REF:0000107
	Neuronal cell body	IEA	UniProtKB:P07824	GO_REF:0000107
	Neuron projection	IEA	UniProtKB:P07824	GO_REF:0000107
	Negative regulation of T cell proliferation	IDA		PMID:16709924
	Cellular response to lipopolysaccharide	IEA	UniProtKB:P07824	GO_REF:0000107
	Extracellular space	IDA		PMID:16709924
	Cellular response to interleukin-4	IEA	UniProtKB:P07824	GO_REF:0000107
CA4	Neuronal cell body	ISS	UniProtKB:P15205	GO_REF:0000024
	Extracellular exosome	IDA		PMID:15326289
	Protein binding	IPI	UniProtKB:Q9Y6R1	PMID:15563508
	Integral component of membrane	IEA	UniProtKB-KW:KW-0812	ZFIN:ZDB-PUB-020723-1
	Regulation of pH	IMP		PMID:16571594
CRISPLD2	Transport vesicle	IDA		GO_REF:0000054
	Extracellular matrix organization	IEA	UniProtKB:Q8BZQ2	GO_REF:0000107
	Heparin binding	IEA	UniProtKB:Q8BZQ2	GO_REF:0000107
	Embryonic viscerocranium morphogenesis	IMP	ZFIN:ZDB-MRPHLNO-130131-3	PMID:26297922
S100A12	RAGE receptor binding	IPI	UniProtKB:Q15109	PMID:15033494
	Positive regulation of I-kappaB kinase/NF-kappaB signaling	IDA		PMID:15033494
	Calcium-dependent protein binding	IBA	PANTHER:PTN007521293	PMID:21873635
	Inflammatory response	IEA	UniProtKB-KW:KW-0395	GO_REF:0000043
	Positive regulation of MAP kinase activity	TAS		PMID:18443896
GZMK	Extracellular region	IEA	UniProtKB-SubCell:SL-0243	GO_REF:0000044
	Protein binding	IPI	UniProtKB:P55061	PMID:32296183

### Identification of miRNAs targeting co-DEGs and functional and pathway enrichment analysis

**Figure 6:**
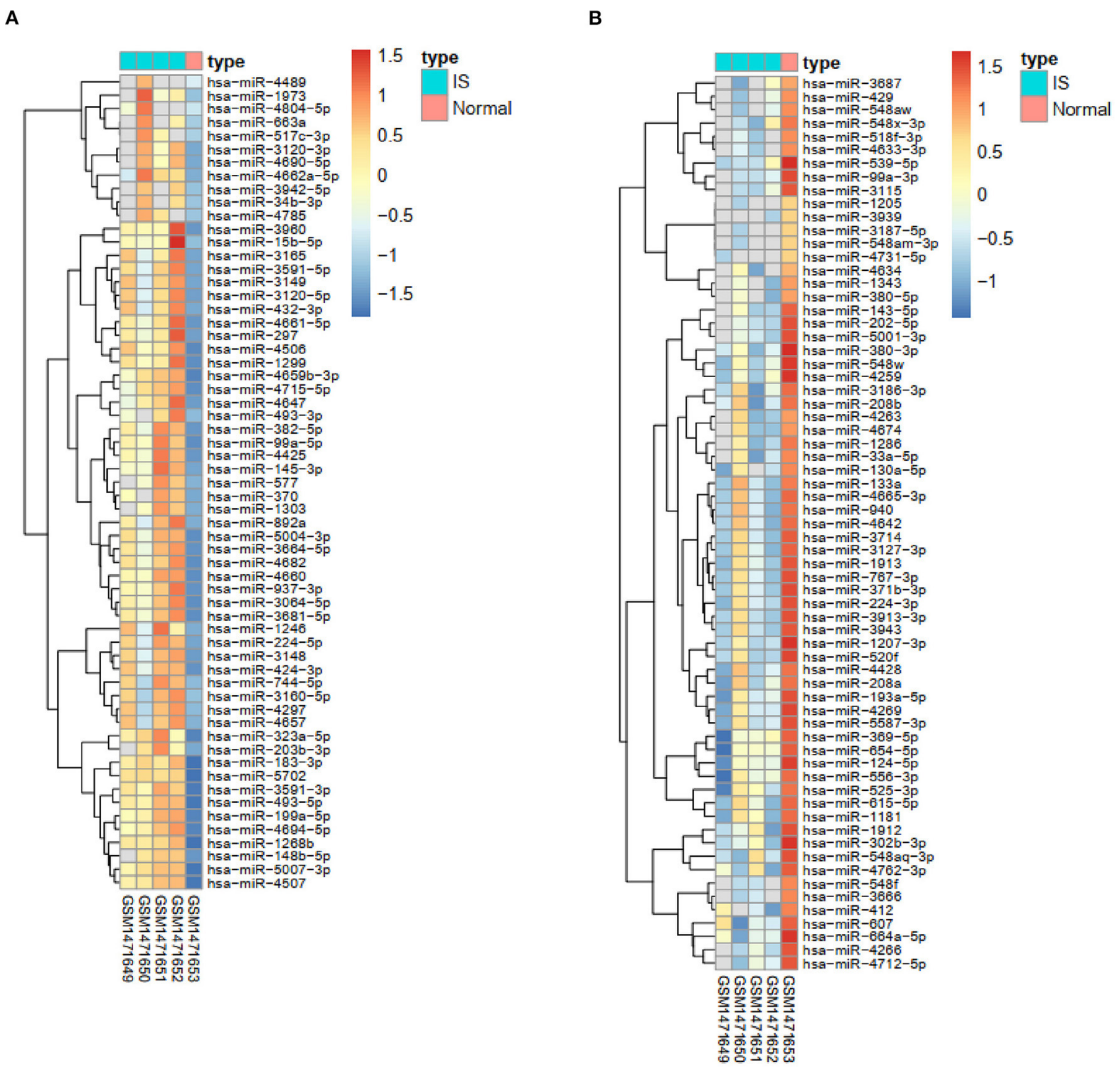
Heatmaps of the expression of DE-miRNAs. **(A)** The heatmap of upregulated IS-specific DE-miRNAs. **(B)** The heatmap of downregulated IS-specific DE-miRNAs.

The TargetScan, mirDIP, miRWalk, and DIANA bioinformatic tools were applied to identify the top five miRNAs targeting each co-DEG for STEMI-related IS (Table 2). In addition, we used the GSE60319 dataset to identify DE-miRNAs in IS and determine the overlap between predicted miRNAs and DE-miRNAs (Figures 1C, 6). (GSE60319: hsa-miR-654-5p, log2FC = −2.67, *p*-value: 0.033).

**Table 2:** GO functional and KEGG pathway analysis of the predicted miRNAs targeting co-DEGs.

**Genes**	**Predicted miRNAs**	**Category**	**Term**	**miRNAs**	***P* value**
ARG1	hsa-miR-1202	KEGG	Hippo signaling pathway	5	1.66E-04
	hsa-miR-340-5p		TGF-beta signaling pathway	4	2.36E-04
	hsa-miR-3692-3p		Phosphatidylinositol signaling system	5	0.017
	hsa-miR-1264		TNF signaling pathway	4	0.017
	hsa-miR-4766-5p		ErbB signaling pathway	5	0.039
		GO	Ion binding	5	1.92E-84
			Neurotrophin TRK receptor signaling pathway	5	7.91E-19
			Epidermal growth factor receptor signaling pathway	5	1.04E-10
			Toll-like receptor signaling pathway	5	3.35E-08
			Fibroblast growth factor receptor signaling pathway	5	1.75E-05
MMP9	hsa-miR-483-3p	KEGG	Fatty acid biosynthesis	3	5.96E-25
	hsa-miR-149-5p		Hippo signaling pathway	4	1.28E-06
	hsa-miR-1224-3p		TGF-beta signaling pathway	4	1.86E-05
	hsa-miR-1306-5p		mRNA surveillance pathway	4	0.010
	hsa-miR-6749-3p		Circadian rhythm	3	0.038
		GO	Response to stress	4	1.78E-08
			Platelet degranulation	4	8.94E-07
			Transforming growth factor-beta receptor signaling pathway	4	0.002
			Thyroid hormone receptor binding	3	0.013
			Cellular response to hypoxia	4	0.013
CA4	hsa-miR-3912-5p	KEGG	Glycosphingolipid biosynthesis - ganglio series	4	1.22E-12
	hsa-miR-204-3p		Thyroid hormone synthesis	5	0.035
	hsa-miR-4747-5p	GO	Ion binding	5	9.94E-25
	hsa-miR-7851-3p		Gene expression	5	4.05E-12
	hsa-miR-671-5p		Neurotrophin TRK receptor signaling pathway	5	3.29E-09
			Synaptic transmission	5	1.35E-05
			Epidermal growth factor receptor signaling pathway	5	3.56E-04
CRISPLD2	hsa-miR-1207-5p	KEGG	Cell adhesion molecules (CAMs)	5	5.03E-05
	hsa-miR-635		Adherens junction	5	0.003
	hsa-miR-634		Axon guidance	5	0.003
	hsa-miR-654-5p		Lysine degradation	5	0.011
	hsa-miR-378a-5p		Morphine addiction	5	0.015
		GO	Neurotrophin TRK receptor signaling pathway	5	1.96E-09
			Response to stress	5	4.40E-04
			Phosphatidylinositol-mediated signaling	5	0.004
			Toll-like receptor signaling pathway	5	0.004
			Regulation of transcription from RNA polymerase II promoter in response to hypoxia	3	0.037
S100A12	hsa-miR-5787	KEGG	Vasopressin-regulated water reabsorption	5	7.70E-06
	hsa-miR-6133		Circadian rhythm	4	0.003
	hsa-miR-6861-5p		TGF-beta signaling pathway	5	0.007
	hsa-miR-5589-5p		Fatty acid elongation	3	0.012
	hsa-miR-5004-5p		Cytokine-cytokine receptor interaction	4	0.038
		GO	Neurotrophin TRK receptor signaling pathway	5	4.89E-06
			Blood coagulation	5	0.005
			Water-soluble vitamin metabolic process	4	0.031
			Apoptotic signaling pathway	5	0.041
			Immune system process	5	0.041
GZMK	hsa-miR-558	KEGG	TGF-beta signaling pathway	4	3.24E-05
	hsa-miR-300		Adherens junction	5	3.24E-05
	hsa-miR-4793-3p		Arrhythmogenic right ventricular cardiomyopathy (ARVC)	4	0.009
	hsa-miR-6088		Axon guidance	4	0.029
	hsa-miR-6741-5p		Insulin signaling pathway	5	0.036
		GO	Protein binding transcription factor activity	5	1.26E-13
			Phosphatidylinositol-mediated signaling	5	3.99E-10
			Fibroblast growth factor receptor signaling pathway	5	1.74E-08
			Nervous system development	5	8.69E-04
			Epithelial to mesenchymal transition	5	0.011

## Discussion

IS is a potential complication of AMI and poses a significant threat to patients (1). Patients with STEMI were found to be more susceptible to having a stroke than the general population (6). The most common confirmed stroke type in patients with STEMI treated with PCI is IS (8). The knowledge gained from identifying genes specifically expressed in STEMI-related IS and the relationships between them may be used to improve the outcomes of patients with STEMI. In this study, we detected that genes involved in the inflammatory and immune response, receptor activity, and protein binding were remarkably related to the maintenance of STEMI and IS occurrence.

Several hub genes regulating the nervous system were observed among the STEMI-DEGs through the analysis of the Comparative Toxicogenomics Database. For example, MMP9, also known as gelatinase B, was found to be an important factor in the occurrence of cardiovascular and nervous system diseases. The Chen's group illustrated that MMP-9 was upregulated in serum exosomes from patients with STEMI, making it a potential biomarker for diagnosis of STEMI (25). Moreover, a higher level of local MMP-9 was observed to be associated with poorer outcomes for patients with STEMI (26). To explore association between MMP-9 and the risk of IS, the Nie's group examined polymorphism of the MMP-9 gene between 400 healthy controls and 396 patients with IS, and found that the MMP-9-1562T allele was associated with an increased risk of IS (27). Another hub gene, ARG1, has been found to be continuously upregulated in patients with acute IS (28, 29). Endocytosis of STAT6/ARG1 can reduce inflammation and improve the outcome of stroke by regulating the phenotypes of macrophages/microglia (30). ARG1 was also observed to be significantly upregulated in patients with AMI and may be used to diagnose AMI (31). Carbonic anhydrase enzymes, which are expressed in mouse and human hearts, are associated with the prognosis of cardiac hypertrophy (32, 33). Although previous studies have illustrated that CA II is the only CA present in the brain, another study observed that CA4 was also located in the mouse brain and may be related to the blood-brain barrier (34). Research into nervous system diseases has identified CA4 as a novel therapeutic target for anxiety disorder and posttraumatic stress disorder (35). The hub gene S100A12 has been shown to have a regulatory role in carotid plaque instability and the occurrence of major cardiovascular events in patients with stable coronary artery disease (36). Furthermore, S100A12 could more accurately diagnose patients with STEMI than other identified biomarkers, and the levels of S100A12 were negatively correlated with the prognosis of IS (37, 38).

Additionally, previous studies have shown that post-treatment with sevoflurane may prevent myocardial ischemia/reperfusion damage through the upregulation of miR-145 and downregulation of GZMK expression (39). Moreover, GZMK was detected to play a significant role in regulating transendothelial cell exudation for central nervous system parenchymal immune surveillance, and it may be an underlying therapeutic target for age-related immune system dysfunction (40, 41). The hub gene CRISPLD2 has been previously found to be a GC and developmental regulatory gene and encodes a mesenchymal protein secreted in the lungs and other organs (42, 43). However, its role in cardiovascular and cerebrovascular diseases is unclear. A recent study has observed that it may be involved in cardiac ischemia/reperfusion injury (44). In addition, CRISPLD2 was found to be associated with several neurodegenerative diseases, but the specific mechanism is not certain (45). Hence, the identities of these hub genes indicate that there may be a potential association between nervous system and cardiovascular disease and that this association may be due to the same pathogenic genes.

It has been widely accepted that miRNA can be used as a biomarker and gene therapy for several diseases. We identified the overlap between predicted miRNAs and DE-miRNAs specific to patients with IS. In particular, hsa-miR-654-5p may be underlying biomarkers of STEMI-related IS. Previous studies have demonstrated that hsa-miR-654-5p is a biomarker of atherosclerosis with an area under the curve (AUC) score of 0.7308 (46). Atherosclerosis is a common pathogenic mechanism of STEMI and IS; hence, hsa-miR-654-5p may be a common therapeutic target. In clinical, the co-DEGs and hsa-miR-654-5p may be served as biomarkers to diagnose IS after patients underwent STEMI. And these co-DEGs may be beneficial to further explore the potential pathophysiological mechanisms between STEMI and IS. Moreover, the co-DEGs may also play an important role in detection of STEMI in patients with IS.

This study is the first data mining to identify co-DEGs between STEMI and IS. Our results give a reasonable speculation for the pathophysiological mechanisms of STEMI-related IS. Our study does have serval limitations. First, our work is a microarray analysis based on different datasets. Hence, the different pieces of clinical information of detected samples in two datasets may have a certain influence on our study. Additionally, validation should be conducted by PCR or Elisa to verify these markers. However, the technique of models for STEMI and IS was immature *in vivo* and *in vitro*. In the future, the larger clinical studies are needed to verify our results to some extent.

## Conclusions

Based on our analyses, the hubgenes CD8A, TLR2, TLR4, S100A12, and TREM1 may be associated with STEMI, and IL7R, CCR7, FCGR3B, CD79A, and ITK may be related to IS. In addition, MMP9, ARG1, CA4, CRISPLD2, S100A12, and GZMK were found to be associated with STEMI-related IS. Lastly, the miRNAs targeting each co-DEG may serve as biomarkers or targets for treatment of STEMI-related IS, especially miR-654-5p.

## Data availability statement

Publicly available datasets were analyzed in this study. This data can be found here: National Center for Biotechnology Information (NCBI) Gene Expression Omnibus (GEO), https://www.ncbi.nlm.nih.gov/geo, GSE60993, GSE16561, and GSE60319.

## Ethics statement

Ethical review and approval was not required for the study on human participants in accordance with the local legislation and institutional requirements. Written informed consent from the patients/participants or patients/participants' legal guardian/next of kin was not required to participate in this study in accordance with the national legislation and the institutional requirements.

## Author contributions

SF and RL were responsible for execution of the research project, data analysis, and writing of the manuscript. QZ and FQ were responsible for execution of the research project. WH and XL were responsible for conception, organization, review, and critique of the manuscript and securing funding. All authors contributed to the article and approved the submitted version.

## Funding

This work was supported by the National Natural Science Foundation of China (U20A20357 and 82001452) and the Fundamental Research Funds for the Central Universities (WK9110000056).

## Conflict of interest

The authors declare that the research was conducted in the absence of any commercial or financial relationships that could be construed as a potential conflict of interest.

## Publisher's note

All claims expressed in this article are solely those of the authors and do not necessarily represent those of their affiliated organizations, or those of the publisher, the editors and the reviewers. Any product that may be evaluated in this article, or claim that may be made by its manufacturer, is not guaranteed or endorsed by the publisher.

## Supplementary material

Supplementary Figure 1 The boxplots of the evaluation of quality of each dataset.

Supplementary Table 1 An Excel spreadsheet listing DEGs of STEMI and IS and co-DEGs.
