# Supplementary figures and images for: Bioinformatics analysis to identify potential biomarkers and therapeutic targets for ST-segment–elevation myocardial infarction-related ischemic stroke
Source: Front Neurol. 2022 Aug 11;13:894289. doi: 10.3389/fneur.2022.894289 (PMC9403764; doi:10.3389/fneur.2022.894289)

GSE60993

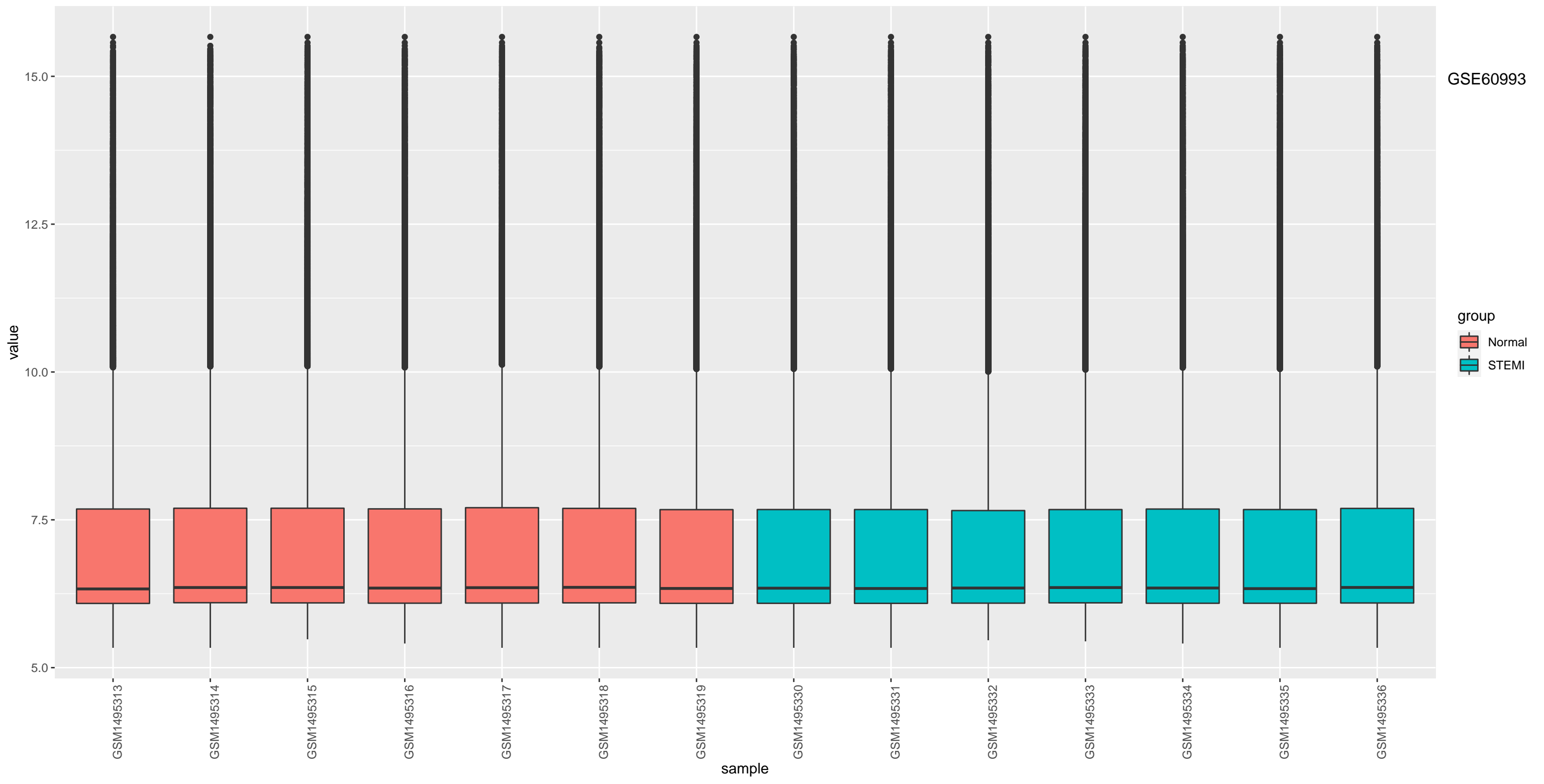

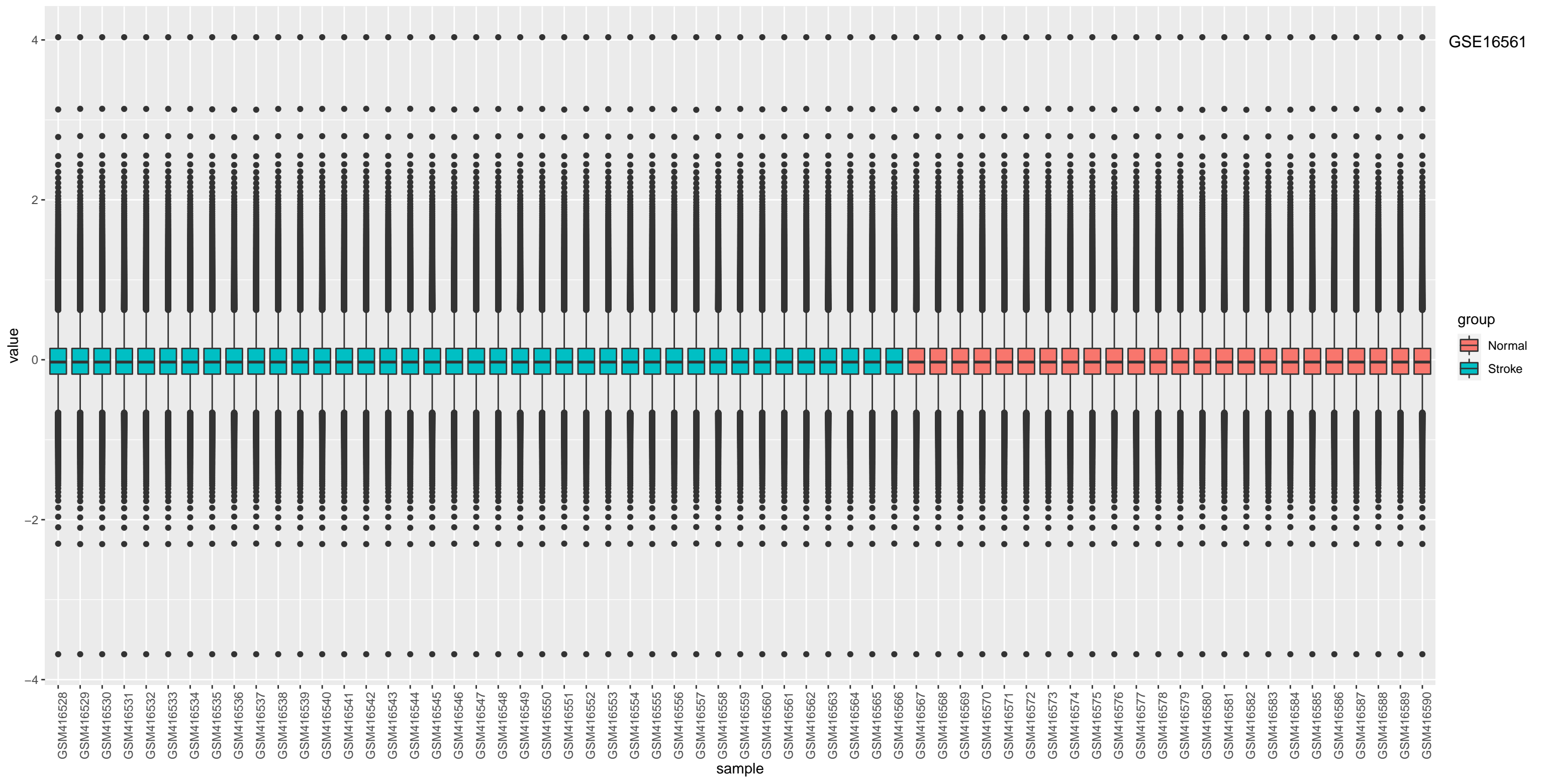

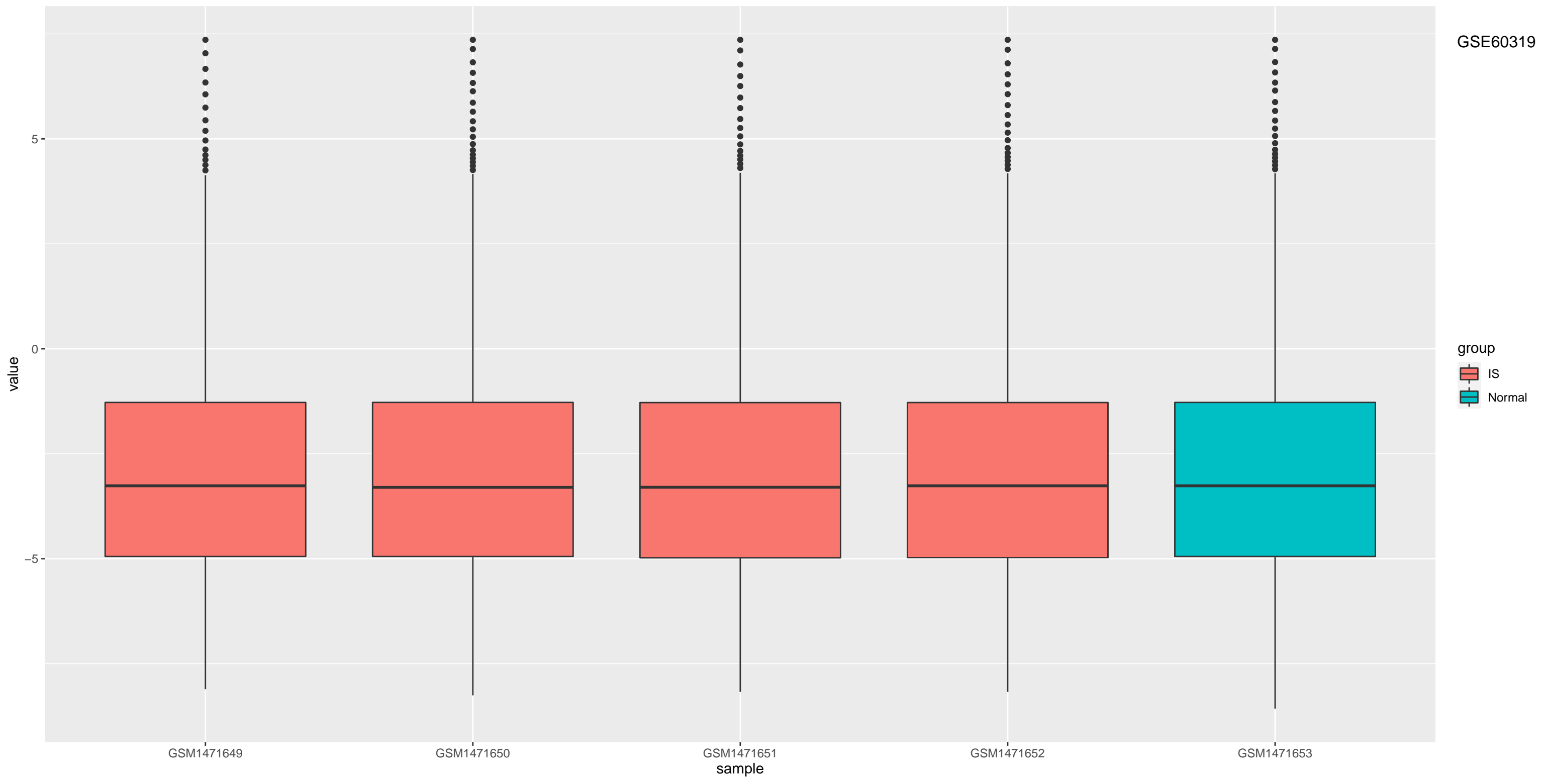

Supplement: Supplementary Figure 1 — The boxplots of the evaluation of quality of each dataset. [file Data_Sheet_1.PDF]
